# Prospective Study of Avian Influenza Virus Infections among Rural Thai Villagers

**DOI:** 10.1371/journal.pone.0072196

**Published:** 2013-08-16

**Authors:** Whitney S. Krueger, Benjawan Khuntirat, In-Kyu Yoon, Patrick J. Blair, Malinee Chittagarnpitch, Shannon D. Putnam, Krongkaew Supawat, Robert V. Gibbons, Darunee Bhuddari, Sirima Pattamadilok, Pathom Sawanpanyalert, Gary L. Heil, Gregory C. Gray

**Affiliations:** 1 College of Public Health and Health Professions and Emerging Pathogens Institute, University of Florida, Gainesville, Florida, United States of America; 2 US Army Medical Component - Armed Forces Research Institute of Medical Sciences, Bangkok, Thailand; 3 Naval Medical Research Unit 2 and Office of Defense Cooperation, Singapore; 4 National Institute of Health, Ministry of Public Health, Bangkok, Thailand; 5 Naval Health Research Center, San Diego, California, United States of America; Arizona State University, United States of America

## Abstract

**Background:**

In 2008, 800 rural Thai adults living within Kamphaeng Phet Province were enrolled in a prospective cohort study of zoonotic influenza transmission. Serological analyses of enrollment sera suggested this cohort had experienced subclinical avian influenza virus (AIV) infections with H9N2 and H5N1 viruses.

**Methods:**

After enrollment, participants were contacted weekly for 24mos for acute influenza-like illnesses (ILI). Cohort members confirmed to have influenza A infections were enrolled with their household contacts in a family transmission study involving paired sera and respiratory swab collections. Cohort members also provided sera at 12 and 24 months after enrollment. Serologic and real-time RT-PCR assays were performed against avian, swine, and human influenza viruses.

**Results:**

Over the 2 yrs of follow-up, 81 ILI investigations in the cohort were conducted; 31 (38%) were identified as influenza A infections by qRT-PCR. Eighty-three household contacts were enrolled; 12 (14%) reported ILIs, and 11 (92%) of those were identified as influenza infections. A number of subjects were found to have slightly elevated antibodies against avian-like A/Hong Kong/1073/1999(H9N2) virus: 21 subjects (2.7%) at 12-months and 40 subjects (5.1%) at 24-months. Among these, two largely asymptomatic acute infections with H9N2 virus were detected by >4-fold increases in annual serologic titers (final titers 1∶80). While controlling for age and influenza vaccine receipt, moderate poultry exposure was significantly associated with elevated H9N2 titers (adjusted OR = 2.3; 95% CI, 1.04–5.2) at the 24-month encounter. One subject had an elevated titer (1∶20) against H5N1 during follow-up.

**Conclusions:**

From 2008–10, evidence for AIV infections was sparse among this rural population. Subclinical H9N2 AIV infections likely occurred, but serological results were confounded by antibody cross-reactions. There is a critical need for improved serological diagnostics to more accurately detect subclinical AIV infections in humans.

## Introduction

After detecting the first highly pathogenic avian influenza (HPAI) poultry outbreaks in 2003 and the first human cases in 2004 in Thailand [1], detections continued until 2006 when intensive bird and human surveillance efforts, poultry culling, poultry vaccination programs, and several other interventions prevented further HPAI transmission [1], [2], [3], [4], [5], [6], [7]. Between 2004–06, 25 human HPAI cases were reported, with a 68% case fatality rate [8]. Infrequent reports of HPAI in domestic poultry continued to be reported in 2007 and 2008 [9], but no poultry illnesses have been reported since 2008.

As influenza surveillance in Thailand is often conducted in urban areas at the best medical facilities [10], [11], people living in rural settings, or people with mild influenza infections who do not seek medical care, may be missed. To better examine the incidence and prevalence of avian influenza transmission in Thailand, adults with poultry exposure living in rural north-central Thailand, as well as their family members, were prospectively followed for 2 yrs for evidence of avian influenza virus (AIV) infections. A previously published report detailed the study methods of enrolling the cohort and presented findings from the serological investigation of enrollment sera [12]. Enrollment data suggested that people in rural central Thailand were experiencing subclinical H9N2 and H5N1 AIV infections as a result of yet unidentified environmental exposures. Lack of an indoor water source seemed to play a role in transmission. We now present prospective data that provides more insight into the seropositivity observed at the time subjects were enrolled in the study.

## Materials and Methods

Details about the study location, study subjects, enrollment methods, database generation, and serology laboratory methods have previously been published [12].

### Ethics Statement

A total of six institutional review boards reviewed and approved the study: University of Iowa; University of Florida; USAMC-Armed Forces Research Institute of Medical Sciences; National Institute of Health, Ministry of Public Health, Bangkok, Thailand; Naval Medical Research Unit No. 2, Jakarta, Indonesia; Human Research Protection Office of the U.S. Army Medical Research and Materiel Command.

All participants signed an informed consent form.

### Weekly Follow-up

During enrollment, cohort participants were given oral and written instructions and a digital thermometer. They were asked to contact study field staff upon developing signs and symptoms of an influenza-like illness (ILI) via a telephone call. Study staff also conducted weekly home visits to remind participants of the importance of reporting ILI and to assess whether an illness was present or had occurred during the preceding week. ILI was defined as acute onset of a respiratory illness with an oral (or equivalent from other body region) measured temperature≥100.5°F (38°C) and a sore throat, cough, shortness of breath, or respiratory distress for 4 or more hours.

### Investigating an Influenza-like Illness

When a possible ILI was reported to study staff, a home visit was performed within 24 hrs of notification. If a focused history confirmed the subject met the ILI case definition, a study nurse completed an ILI questionnaire and collected an acute serum sample and 2 respiratory swab specimens (nasal and pharyngeal). The swab specimens were stored in viral transport media and sent on wet ice to the Kamphaeng Phet-AFRIMS Virology Research Unit (KAVRU) located at the study site. Sixty days following the ILI investigation, study staff returned to the subject's home to collect a convalescent serum sample. If a participant developed a second case of ILI during the convalescent period that the site principal investigator judged to be distinct from the original illness, the second ILI episode was considered a unique event and a new investigation was initiated.

### Family Transmission Study

A family investigation was initiated when a consented, enrolled cohort study participant developed an acute influenza A infection confirmed by real-time RT-PCR (qRT-PCR). This visit was scheduled with the family as soon as possible after the cohort subject was found to have an influenza A infection (within 1–2 days). During the home visit, members who lived in the same household (defined as ≥20 days per month under the same roof) were invited to participate. Informed consent was obtained from any family member who wished to participate. Parents or guardians signed for family members <20 years of age. In addition, family members who were between 7 and 20 years of age and who wished to participate signed an assent form.

Study staff completed an ILI Case Household Form (one per household) through an interview with an adult household member. In addition, each consenting family member completed an ILI Case Contact Form to assess the individual's contact with the ill cohort member as well as their recent animal exposures. Study staff collected an acute serum sample from each consenting subject. If a family member met the ILI case definition, a nasal and pharyngeal swab were also collected at the time of his/her enrollment. Family members were visited weekly for 9 weeks to monitor their possible development of ILI. If a family member met the ILI case definition, then respiratory swab specimens were collected. Sixty days following family members' enrollment, study staff returned to the home to collect a convalescent serum sample.

### Annual Follow-up

Twelve and 24 months following enrollment, study participants completed annual follow-up visits. Similar to enrollment procedures [12], participants provided a serum sample and completed a follow-up questionnaire that assessed any changes to their demographics, health, or animal exposures in the past year. Serological analyses of the annual sera were performed to monitor changes in influenza antibody titers over time.

### Replacement Enrollments

In order to maintain the number of active cohort subjects at around 800 participants, a replacement subject was enrolled after a cohort subject withdrew from the study for any reason. Study staff recruited the replacement subject from the non-enrolled household physically closest to the household from which the withdrawn subject came. Study staff randomly enrolled one adult from the replacement household following a similar process as the initial enrollment [12]. If all adults in that household refused to participate in the study, then study staff went to the next nearest household and continued in this way until an adult replacement cohort member was enrolled. After enrollment, weekly follow-up as well as other activities (i.e. ILI investigation, family transmission study, etc.) began for that individual.

### Laboratory Methods

Each respiratory specimen from prospective cohort subjects meeting the ILI case definition and from family member contacts were tested with qRT-PCR for influenza A at the KAVRU laboratory, within 72 hours of collection. Swab specimens (1.0 ml) were first warmed to room temperature. Viral RNA was extracted from 140 µl of the specimen and processed using the Qiagen: QIAamp Viral RNA Mini Kit (Qiagen Inc., Valencia, California) following a mini-spin protocol. Contaminants were washed away by two wash buffers and the RNA eluted in 50 µl of elution buffer.

A qRT-PCR strategy similar to that described by Fouchier et al. [13] that detects a conserved region of the influenza matrix gene was used to screen for all influenza A viruses. When positive, as per World Health Organization guidelines, the specimen was further evaluated with qRT-PCR assays against human H1, human H3, avian H5, and swine H1 influenza viruses. Thermocycling was performed using a Rotor-Gene RG-3000 thermocycler or similar real-time PCR equipment. All qRT-PCR runs included a template negative control and the corresponding primer set viral template positive control. Each extraction run included a mock extraction control to provide a secondary negative control to validate the extraction procedure and reagent integrity. The human RNase P gene primer set was used as an internal positive control for human RNA in each sample. Specimens that were qRT-PCR positive for generic influenza type A were further evaluated with a qRT-PCR procedure specific for H1, H3, and H5 [14].

To validate the molecular results, qRT-PCR positive swab samples were also cultured for virus isolation and characterization by the Thailand Ministry of Health. Briefly, respiratory swabs in viral transport media were centrifuged at 8,000 rpm for 20 minutes at 4°C. Using the supernatant as the inoculum material, 0.2 ml was added to each 25 cm^2^ flask of confluent Madin-Darby Canine Kidney (MDCK) cells (2 cell flasks were inoculated per swab). Flasks were incubated at 37°C±1°C for 1 hr, with rocking every 15 minutes to distribute the inoculum. After one hour, 5 ml of maintenance medium was added to each flask and incubated at 35°C±1°C. Each flask was observed daily microscopically for 7–10 days for cytopathic effect (CPE). If CPE was observed, cells were harvested for immunofluorescence assay (IFA) staining with a FITC conjugated influenza A specific monoclonal antibody. For flasks with no CPE after 10 days, the culture medium was aspirated and 0.2 ml used to inoculate second passage following the procedure described above. If no morphological change was observed after 10 days in the second passage, and the negative result was confirmed by IFA, the swab was considered as culture negative and no further testing was performed. If CPE was observed during the second passage, the harvested cells were further tested by IFA using specific monoclonal antibody as described above.

Serological studies were performed at the University of Florida's Global Pathogens Laboratory and the Thailand National Institute of Health. Serological analyses of 12- and 24-month follow-up sera were performed using a previously described HI assay [15] to test for serum antibodies against 3 human and 3 swine influenza A viruses (Table 1). A MN assay adapted from that reported by Rowe [16], [17], [18] was used to detect antibodies against a panel of 10 avian and avian-like influenza viruses (Table 1). Paired ILI sera were tested for antibodies against the 3 human influenza viruses, as well as A/Thailand/384/2006(H5N1), A/Thailand/676/2005(H5N1), and A/Hong Kong/1073/99(H9N2).

**Table 1:** Viruses used in serological studies. Unless otherwise indicated serologic study was performed using the microneutralization technique.

Avian viruses	Swine viruses
A/Migratory duck/Hong Kong/MPS180/2003(H4N6)	A/Swine/Lutol/3/2000(H1N1)^a^
A/Nopi/Minnesota/07/462960-2(H5N2)	A/Swine/Gent/7625/1999(H1N2)^a^
A/Teal/Hong Kong/w312/1997(H6N1)	A/Swine/Flanders/1/1998(H3N2)^a^
A/Env/Hong Kong/MPB127/2005(H7N7)	
A/Migratory duck/Hong Kong/MP2553/2004(H8N4)	Human viruses
A/Migratory duck/Hong Kong/MPD268/2007(H10N4)	A/Brisbane/59/2007(H1N1)^a^
A/Chicken/New Jersey/15906-9/1996(H11N1)	A/Mexico/4108/2009(pandemic H1N1)^a^
A/Duck/Alberta/60/1976(H12N5)	A/Brisbane/10/2007(H3N2)^a^
	A/Thailand/384/2006(H5N1)b,c
	A/Thailand/676/2005(H5N1)b,c
	A/Hong Kong/1073/99(H9N2)^b^

a Virus studied with hemagglutination inhibition assay.

b Virus of avian origin.

c Highly pathogenic virus, Clade 1.

### Statistical Methods

Study outcomes were evidence of previous or acute influenza A virus infections. Acute influenza infection was defined as either a) isolation of influenza virus from a respiratory specimen obtained when a patient had an influenza-like illness, b) qRT-PCR evidence of influenza from such specimens, or c) a fourfold or greater rise in antibody titer against an influenza virus for paired ILI or annual follow-up sera. Because serologic responses to AIV infection can rapidly wane [19], as we have reported previously [12], [20], we chose a low threshold of antibody titer (≥1∶10) as evidence of previous infection with an AIV strain. Because we know that cross-reactions from previous infection with human viruses might confound avian influenza virus serology, we sought to control such potential confounding by adding human influenza virus reactivity covariates to the multivariate models when the bivariate analyses suggested they were important outcome predictors. As done previously [12], [18], [21], [22], [23], a HI titer ≥1∶40 was accepted as evidence of human or swine influenza virus infection or human influenza vaccination.

Initially we examined risk factors for bivariate associations with MN assay results using binary logistic regression and proportional odds modeling [24]. An exact method was used for sparse data, and the score test was used to evaluate the proportional odds assumption. Covariates with p values <0.25 were considered for inclusion in multivariate models. Final multivariate models were designed using manual backwards elimination. Analyses were performed by using SAS v9.2 (SAS Institute, Inc., Cary, NC, USA).

## Results

Between April and October 2008, field staff enrolled a total of 800 participants (100 from each of 8 sites). Participant demographics at enrollment have previously been reported [12]. Over the 24-month follow-up period, 49 subjects withdrew their participation and 45 replacement enrollments were added. A total of 768 participants (96%) completed the 12-month annual follow-up and 784 participants (98%) completed the 24-month annual follow-up visit. Overall, 747 participants (93%) remained enrolled for the entire study duration by completing enrollment and both 12- and 24-month follow-up visits. Eighty-one ILI investigations were conducted among 74 cohort subjects (6 subjects experienced >1 unique ILI event) and 83 household contacts were enrolled in the family transmission study with 12 contacts (14%) developing ILI.

### Acute Human Influenza A Infections

**Figure 1:**
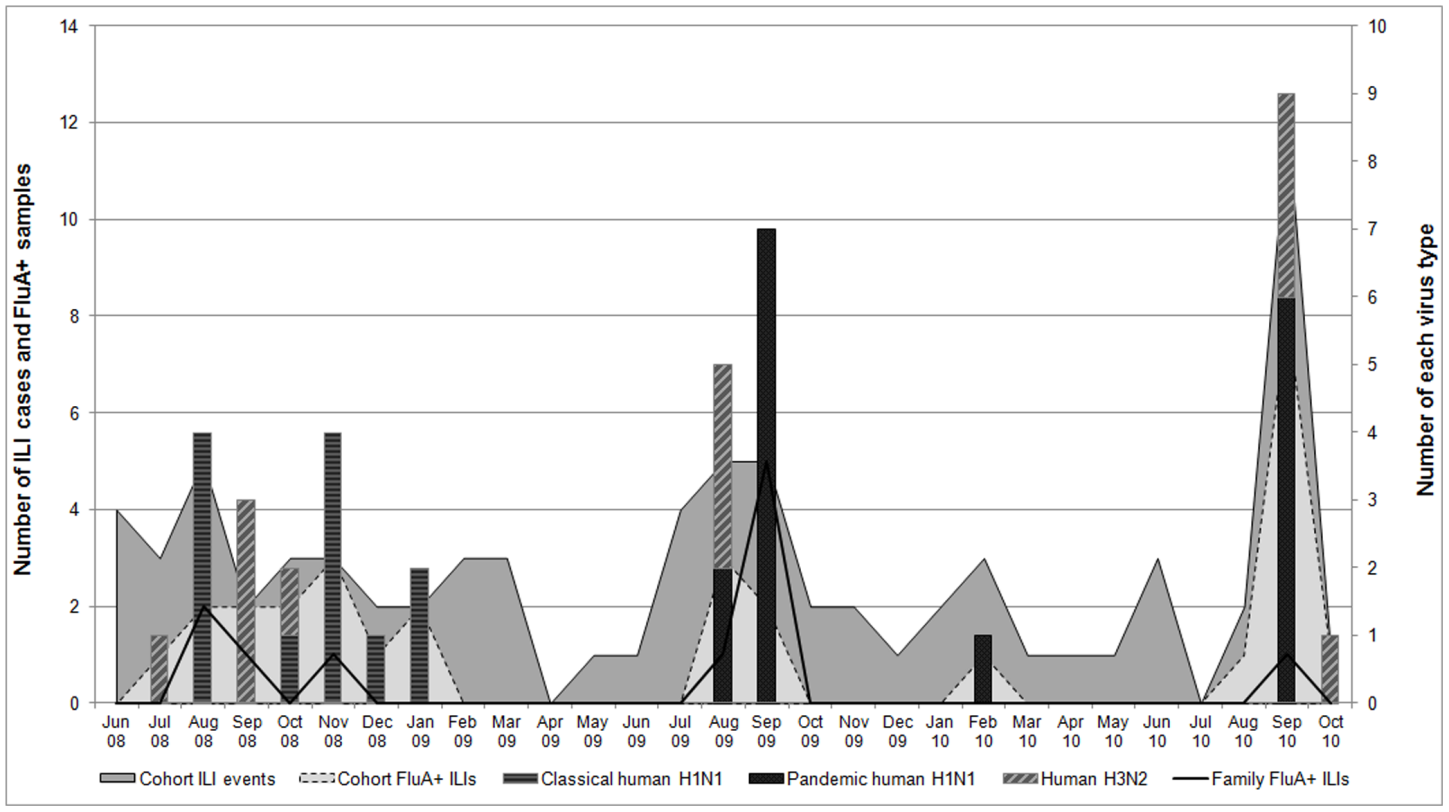
Reported influenza-like illnesses with real-time RT-PCR results of respiratory swabs collected from study cohort members and their family contacts at time of illness; June 2008–November 2010; Kamphaeng Phet, Thailand.

qRT-PCR analyses were performed on nasal and pharyngeal swabs collected during ILI episodes (Figure 1). For the cohort subjects, 31 (38%) of the 81 reported ILIs were qRT-PCR-positive for influenza A virus from the nasal and/or pharyngeal swab; of the 12 ILI cases among family contacts, 11 (92%) were qRT-PCR positive for influenza A. Among the 42 total participants positive by qRT-PCR for influenza A, 12 (9 cohort members, 3 contacts) had the classical human H1N1 influenza A virus, 16 (10 cohort members, 6 contacts) had influenza A(H1N1)pdm09 virus, and 13 subjects (11 cohort members, 2 contacts) were positive for human H3N2 influenza A virus. In addition, one ill cohort member had a dual infection by qRT-PCR with a H3N2 influenza A virus and an influenza B virus. Positive qRT-PCR results were confirmed by IFA staining the cultures of respiratory swabs inoculated on MDCK cells. qRT-PCR and IFA results were in agreement for 80% of the swabs; the remaining 20% of swabs failed to produce a positive influenza A culture by IFA.

Incident influenza A infections were also defined by a 4-fold or greater increase in HI antibody titers between acute and convalescent sera. For the 43 subjects (out of 74 total symptomatic subjects) who experienced an ILI but were negative for influenza by qRT-PCR, 7 (16%) had serological evidence of influenza infection: 1 for H1N1 and 6 for H3N2. As paired sera were collected from all household contacts, regardless of experiencing ILI symptoms, 27 (38%) of the 71 largely asymptomatic family members experienced a ≥4-fold increase in antibody titers from the acute to convalescent blood draws, yet never reported experiencing symptoms consistent with ILI. When examining corresponding serological reactivity of the 42 influenza positive qRT-PCR results, 1 (8%) of the 12 subjects qRT-PCR positive for classical H1N1 had a corresponding increase in HI titer; 10 (63%) of 16 subjects qRT-PCR positive for pH1N1 had a ≥4-fold increase in antibody titer, while 7 (50%) of the 14 subjects positive for H3N2 had a ≥4-fold titer increase.

Numerous subclinical or mild influenza A virus infections not detected as ILIs were identified through ≥4-fold increases in antibody titers amongst the cohort by analyses of annual follow-up sera. An additional 312 subjects had evidence of infection with classical human H1N1, of which 301 (96%) did not report an ILI during the respective follow-up period. Of the 11 cases that did report an ILI, none were qRT-PCR positive for H1N1. A total of 275 subjects' titers increased ≥4-fold against human H3N2 influenza over the annual follow-up periods; 264 (96%) had no corresponding report of ILI during the respective follow-up time. Of the 11 who reported ILIs, 3 subjects (27%) were also qRT-PCR positive for H3N2. Against the pandemic H1N1 influenza virus, annual antibody titers increased ≥4-fold between annual bleeds among an additional 124 subjects; 115 did not report an ILI (93%). Of the 9 who experienced an ILI, 3 were qRT-PCR positive for pH1N1.

### Avian Influenza virus Infections

No AIV infections were detected by molecular analyses of the respiratory swabs nor by serological analyses of the paired ILI sera. Contrasting from the sera collected at the time of enrollment [12], there was little serological reactivity against the HPAI H5N1 viruses among the subjects sampled 12 and 24 months after enrollment. Only one subject had an elevated titer (1∶20) against A/Thailand/676/2005(H5N1) at the 24-month follow-up visit; the subject's sera collected previously at the time of study enrollment and at 12-months were not seropositive for H5N1.

Serological reactivity against low-pathogenic avian influenza (LPAI) viruses was also sparse, with the exception of A/Hong Kong/1073/1999(H9N2). At the 12-month follow-up, 21 subjects had elevated antibody titers against this avian-like H9N2 influenza virus: 10 (1∶10), 8 (1∶20), and 3 (1∶80). Two of the participants with 1∶80 titers experienced a >4-fold increase in titer compared to their enrollment sera. At the 24-month follow-up, 40 subjects had elevated titers against this virus: 21 (1∶10), 13 (1∶20), 5 (1∶40), and 1 (1∶160). Five participants had elevated H9N2 titers at all 3 encounters; 16 subjects had elevated titers at both the 12- and 24-month follow-up visits. In bivariate analyses, prior receipt of a human influenza vaccine was significantly associated with elevated antibodies against H9N2 for both 12- and 24-month follow-ups ([OR = 11.4, 95% CI, 2.5–41.2] and [OR = 5.0; 95% CI, 1.9–12.3], respectively). In multivariate analyses of the 40 subjects seropositive at the 24-month follow-up encounter, while controlling for age and influenza vaccine receipt, moderate poultry exposure (7–20 birds/day) in the past 12 months was significantly associated with elevated H9N2 titers (adjusted OR = 2.3; 95% CI, 1.04–5.2) (Table 2). Other LPAIs with low serological reactivity included the H6N1 virus (1 subject at 1∶20 at 12-months), H7N7 virus (1 subject at 1∶40 at 24-months), and H12N5 virus (2 positive subjects with titers at 1∶40 and 1∶320 at 24-months). The subject with the 1∶40 titer against H12N5 also had an elevated titer (1∶40) against the avian H9N2 virus. Among the 49 subjects with elevated titers against AIVs, the median age was 57 yrs, with an interquartile age range of 51–63 yrs. Twenty-five (51%) were female.

**Table 2:** Risk factors for elevated antibodies against A/Hong Kong/1073/1999(H9N2), among adult participants, Thailand, 2010 (24-month follow-up visit) using binary logistic regression^a^.

Variables	Total N	N (%)	Unadjusted OR (95% CI)	Adjusted OR (95% CI)
Age(yrs)				
* (continuous)*	782	40 (100.0)	**1.03 (1.004–1.1)**	**1.03 (1.002–1.1)**
Gender				
Male	318	20 (50.0)	1.5 (0.8–2.8)	–
Female	464	20 (50.0)	Ref	–
A/Brisbane/10/2007(H3N2)^b^				
Positive	242	23 (57.5)	1.7 (0.9–3.2)	–
Negative	540	17 (42.5)	Ref	–
Indoor water				
No	255	10 (25.0)	0.7 (0.3–1.4)	–
Yes	527	30 (75.0)	Ref	–
Received an influenza vaccination in last 12 months^c^				
Yes	44	8 (20.5)	**5.1 (2.2–11.8)**	**5.5 (2.3–13.1)**
No	737	31 (79.5)	Ref	Ref
Exposed to poultry in last year				
21–12,000 birds/day (mean: 933 birds)	120	5 (12.5)	0.9 (0.3–2.4)	1.1 (0.4–3.0)
7–20 birds/day (mean: 14 birds)	116	10 (25.0)	2.0 (0.9–4.3)	**2.3 (1.04–5.2)**
1–6 birds/day (mean: 4 birds)	67	3 (7.5)	1.0 (0.3–3.3)	0.9 (0.3–3.3)
No	479	22 (55.0)	Ref	Ref

a Binary logistic regression (Negative = H9N2 titer <1∶10, Positive = H9N2 titer ≥1∶10).

b H3N2 antibody titer: Negative = titer <1∶40, Positive = titer ≥1∶40.

c Covariate has missing data.

There was a high prevalence of serological reactivity against the 3 swine influenza viruses (SIVs); however, less than 50 respondents reported any swine exposure. This seroreactivity was likely a reflection of cross-reactivity due to human influenza virus infection.

### Attack Rates

Overall, 525 cohort subjects experienced at least one acute human influenza infection during the 2 yr follow-up period, for a crude primary attack rate (PAR) of 65.6%; specifically 38 were symptomatic cases (4.8% PAR) and 487 subjects experienced subclinical or mild infections (60.9% PAR). Interestingly, the annual PAR varied considerably between the 2 yrs of follow-up. The annual PAR for year 1 was 10.5%, while the annual PAR for year 2 was 59.8%.

Among the cohort's 83 family contacts, for the duration of 9 weeks of each case contact monitoring, 38 family members had qRT-PCR or serologic evidence of acute influenza infections for an overall crude secondary attack rate (SAR) of 45.8% for the study period. Eleven family members experienced qRT-PCR-confirmed symptomatic human influenza infections, for a 13.3% symptomatic SAR. Annually, the SAR was 19.3% for year 1, while the annual SAR increased to 26.5% for year 2.

## Discussion

Results of this prospective follow-up study of 800 poultry-exposed, rural Thai participants and their family members suggest that AIV infections were sparse among this population during the 2 yr study period. Clinical illnesses associated with AIV infections were not detected over the 2 yrs of weekly ILI monitoring. Annual blood draws to serologically detect subclinical or mild AIV infections showed little evidence of new infections against 10 avian or avian-like influenza viruses except for H9N2. However, antibodies acquired through subclinical AIV infections likely wane within one year [19]; testing of only 12- and 24-month annual sera may have missed infections. Two subjects' 12-month follow-up sera did have a ≥4-fold antibody titer increase against H9N2 and MN titers of 1∶80. These subjects did not report an ILI during the corresponding follow-up period, suggesting possible subclinical human infections with H9N2. Seroreactivity against the avian-like H9N2 strain was significantly associated with prior receipt of human influenza vaccines. When controlling for age and influenza vaccinations, moderate poultry exposure during the 12 months prior to the 24-month blood draw was significantly associated with elevated antibody titers against H9N2 AIV, suggesting that despite confounding antibody cross-reactivity, some of the H9N2 seropositivity reflected true infections associated with poultry exposure. It was interesting to note that only moderate poultry exposure was associated with elevated titers; the highest ordinal level of poultry exposure (≥21 birds/day) was not associated with elevated titers, compared to subjects who reported no exposure to poultry during the prior 12 months. We hypothesize that cohort members exposed to poultry in larger domestic farms benefited from better biosecurity at these larger businesses.

As persons exposed to viruses from the 1956-8 H2N2 influenza pandemic might have cross-reacting antibodies against H9N2 viruses, we examined age for those subjects with H9N2 reactivity. While the median age of cohort was 48 (born in 1960), subjects born on or before 1968 (H2N2 stopped circulating) were not significantly more likely to have elevated titers against the avian-like H9N2 virus than were subjects born afterwards (OR = 2.2; 95% CI, 0.9–5.8).

Figure 1 illustrates the seasonal trend for qRT-PCR-confirmed human influenza A infections, as well as the introduction of pH1N1 infections in August 2009 that completely replaced the previously circulating classical H1N1 strain. Illness peaks associated with pH1N1 infections seen at August-September 2009 and September 2010 are very similar to those identified by two other epidemiological surveillance studies conducted in Thailand [25], [26].

One explanation for the difference between primary attack rates for year 1 and year 2 may be that classical H1N1 predominated during year 1 and then disappeared in year 2 at the emergence of pH1N1. The PAR for year 1 may have been substantially underestimated if serology is not as robust at detecting classical H1N1 infections, compared to pH1N1 and H3N2 infections. The World Health Organization estimates the global annual influenza attack rate at 5–10% for adults and 20–30% for children [27]. A study of the pH1N1 attack rate in Hong Kong between April-December 2009 reported an overall attack rate of 10.7%. After the first pH1N1 wave, a 2009 serosurvey conducted in Bangkok, Thailand estimated the pH1N1 infection rate in the general population to be 3.1% in adults and 58.6% in children [28].

The WHO estimates annual seasonal influenza SARs to range from 5% to 15%, and SARs for pH1N1 to range from 22%–33% [29], which are similar to the SARs reported in this study. A 2009 Hong Kong study of secondary attack rates for pandemic and seasonal influenza virus infections monitored family members of qRT-PCR positive index patients seeking care at outpatient clinics in July and August [30]. After following contacts for 7 days, they reported a SAR of 8% (95% CI, 3–14) and 9% (95% CI, 5–15) for pandemic and seasonal influenza virus infections, respectively.

Influenza virus infections were confirmed in 38 (46.9%) of the 81 reported cases of ILI among the study cohort. This suggests that other pathogens were responsible for approximately half of the illnesses observed in the study population. However, some have argued that the conventional serological marker of infection, a 4-fold or greater antibody titer increase between acute and convalescent sera, may be underestimating attack rates and by using a data augmentation model, a 2-fold titer change may provide sufficient evidence of infection for population-based data (i.e. not in terms of diagnosing individual cases) [31]. Unfortunately, the study's focus was limited to influenza viruses so we were not able to determine the contribution of other pathogens to disease incidence.

Our findings of sparse evidence for non-H9N2 AIV infections in this cohort during the study period may be due to better control of AIV infections in Thai poultry since control measures were implemented in the mid-2000’s. It is possible that the H5N1 seroreactivity observed among the 2008 enrollment sera were due to residual declining titers from exposure prior to 2008 which became undetectable in 2009. However, animal surveillance reports do suggest that AIVs continue to circulate to some degree among poultry in Thailand. LPAI H4N6 and H4N9 viruses were detected at Thai bird markets in 2009 [32]. In addition, duck serosurveillance conducted by Beaudoin et al in 2010 found serological evidence that H5 influenza A viruses were still circulating among free-grazing ducks [33]. Therefore, our lack of molecular or serological evidence of human infections with AIVs between 2008–2010 is likely to be a result of limited AIV transmission among their often rural, backyard poultry farms. While no signs of ILI among participants' birds were ever reported by study participants, an active surveillance effort of AIV carriage among the participants' flocks would have been a useful added strategy in understanding transmission dynamics.

One limitation of the study was restricting eligibility for the main cohort members to only include adults at least 20 years of age. While this was done for multiple reasons, this decision excluded a large at-risk sub-population [34], [35]. The rates of symptomatic and subclinical/mild infection are likely different in adults compared to young children, as well as the pre-existing immune status and consequent impact on infection rates, clinical presentation, and serological assays. In addition, the initial case in the household may have already occurred (most likely in a child) by the time we initiated a household transmission investigation. Nevertheless, as a part of the active surveillance efforts to detect ILIs, children were included in our family transmission study; however, no evidence of AIV infections among contact children was detected.

Another possible limitation of the study was the specificity of our serological assays. We may have missed evidence of subclinical AIV infections, as well as evidence of clinical human influenza infections by serology, if the viruses used in the laboratory assays were different than the virus strains circulating in Thailand. Efforts were made to match the serological assays with known viruses circulating in Thailand as much as possible. In addition, cross-reacting antibodies resulting from human influenza vaccinations or naturally-acquired human influenza virus infections likely confounded some of our seroreactivity seen against the H9N2 AIV. There is a critical need for improved serological diagnostics to more accurately detect novel AIV infections in humans.

This was a difficult and resource-intensive study to execute. It required numerous field workers, dedicated field laboratory and data entry staff, and thousands of hours of post-collection serological analyses and complex data analyses. It is one of the few large, tightly executed prospective cohort studies of avian influenza transmission. While it yielded the desired PAR and SAR statistics for human-adapted influenza A viruses in this rural population, it was unable to prospectively detect a large number of AIV infections. It appears that to do so would require studies of considerably larger scale and more resources. Such studies may in most circumstances be cost-prohibitive.
